# Explaining Dog Wolf Differences in Utilizing Human Pointing Gestures: Selection for Synergistic Shifts in the Development of Some Social Skills

**DOI:** 10.1371/journal.pone.0006584

**Published:** 2009-08-28

**Authors:** Márta Gácsi, Borbála Gyoöri, Zsófia Virányi, Enikö Kubinyi, Friederike Range, Beatrix Belényi, Ádám Miklósi

**Affiliations:** 1 Department of Ethology, Eötvös University, Budapest, Pázmány, Hungary; 2 Konrad Lorenz Institute for Evolution and Cognition Research, Altenberg, Austria; 3 Department of Neurobiology and Cognition Research, University of Vienna, Vienna, Austria; 4 Wolf Science Center, Grünau im Almtal, Austria; Indiana University, United States of America

## Abstract

**Background:**

The comparison of human related communication skills of socialized canids may help to understand the evolution and the epigenesis of gesture comprehension in humans. To reconcile previously contradicting views on the origin of dogs' outstanding performance in utilizing human gestures, we suggest that dog-wolf differences should be studied in a more complex way.

**Methodology/Principal Findings:**

We present data both on the performance and the behaviour of dogs and wolves of different ages in a two-way object choice test. Characteristic behavioural differences showed that for wolves it took longer to establish eye contact with the pointing experimenter, they struggled more with the handler, and pups also bit her more before focusing on the human's signal. The performance of similarly hand-reared 8-week-old dogs and wolves did not differ in utilizing the simpler proximal momentary pointing. However, when tested with the distal momentary pointing, 4-month-old pet dogs outperformed the same aged hand reared wolves. Thus early and intensive socialisation does not diminish differences between young dogs and wolves in behaviour and performance. Socialised adult wolves performed similarly well as dogs in this task without pretraining. The success of adult wolves was accompanied with increased willingness to cooperate.

**Conclusion/Significance:**

Thus, we provide evidence for the first time that socialised adult wolves are as successful in relying on distal momentary pointing as adult pet dogs. However, the delayed emergence of utilising human distal momentary pointing in wolves shows that these wild canines react to a lesser degree to intensive socialisation in contrast to dogs, which are able to control agonistic behaviours and inhibition of actions in a food related task early in development. We suggest a “synergistic” hypothesis, claiming that positive feedback processes (both evolutionary and epigenetic) have increased the readiness of dogs to attend to humans, providing the basis for dog-human communication.

## Introduction

Recent interest in the evolution of social cognitive abilities in animals puts the domestic dog at the forefront of research [1]. For example, comparative research provided evidence on the outstanding ability of dogs to rely on human pointing gestures, even when the pointing hand was distant from the signalled location, and could not be seen while the animal made its choice (‘distal momentary pointing’, see [2] for review). Dogs even have been found to perform better in some human related communicative tasks compared to chimpanzees [2], [3].

The origin of this skill of dogs has mainly been discussed from an evolutionary point of view. Originally it was hypothesized that selection during domestication might have directly facilitated human-compatible social cognition in dogs [3], [4]. A more recent hypothesis has argued for indirect selection as an alternative explanation. It suggests that in dogs, selection for decreased “emotional reactivity” led to lower levels of fear and aggression, and higher interest and contact seeking towards humans, which in turn enabled canid cognitive skills to be applied in interspecific interactions [5]–[7]. More recently, one study found that intensive socialisation and regular training of wolves diminish some of the previously suspected differences in social cognitive skills between dogs and wolves. This led the authors to emphasise the contribution of ontogenetic effects on the emergence of social skills in socialised dogs and wolves [8].

Unfortunately, broadly presented theories have not always been supported by experimental data or the experimental procedures employed can be criticized [e.g. 9]. For example, the claim about selection for decreased emotional reactivity has not been tested by behavioural observations in socialized wolves and dogs, nor has fearful or aggressive resistance to interaction with humans been shown to correlate at the individual level with the actual performance in communicative tasks [5]. Similarly, there is little data on the social cognitive abilities of adult wolves, which would be necessary to investigate the effect of individual experiences [3], [4], [10]. Unfortunately, results of the two studies on adult wolves available so far [3], [8] cannot be used for comparisons with earlier developmental data [11], [12], because simpler versions of the pointing gesture were used. The study of Udell et al. [8] tested adult wolves with distal pointing; however, in contrast to the definition for momentary pointing [see 13] the pointing gesture was still visible when the subjects made their choice. Moreover, Udell et al. applied a special training technique involving a secondary reinforcer (clicker-training), which probably affected the performance.

Reviewing all these non-exclusive hypotheses we offer a “synergistic” hypothesis that may help in disentangling the main factors, which contribute to the differential communicative performance toward humans in dogs and wolves. Beyond the actual cognitive ability for relying on human directional signals these factors seem to have two main origins.

Anthropogenic selective environment affected probably the mode of action in dogs by changing emotionality and reactivity to stimulation [for reviews see 6], [14] in comparison to their ancestors. As a consequence dogs are generally predisposed to develop better skills for action inhibition that in a social context results in higher willingness for cooperation with humans.

Independently, selection has affected behavior systems dealing with the recognition of social partners and the minimally required amount of socialization. Thus it is expected that dogs exhibit epigenetically enhanced sensitivity for salient human communicative cues. This is supported by differential attachment to humans in similarly socialized dogs and wolves [15], and social environment-dependent variability in sensitivity to human communicative cues in wolves [8], [3], [12]. We suggest that positive feedback between evolutionary (selective) and ontogenetic processes contributed to the increased readiness of dogs to look at the human face providing the basis for complex forms of dog-human communication [11], [16].

Here we present new data which may enhance the plausibility of the synergistic hypothesis. We have tested socialized wolves and pet dogs at three different ages in a two-way object choice task in order to reveal what kind of species-specific differences emerge and how they change over development. For this purpose, in addition to the animals' success in relying on human pointing, we also recorded behavioural indicators of reactivity and emotionality (willingness to cooperate with the humans; aggression, struggling against being restrained) and attention paid to the human. By analysing these behaviours, we investigated whether success in using human pointing changes during development in parallel with the willingness to be controlled and cued by humans, and whether there is a correlation between these factors at the individual level.

## Methods

No special permission for use of animals (wolves) in such socio-cognitive studies is required in Hungary or in Austria. The relevant committees that allow to run research without special permissions regarding animals are: University Institutional Animal Care and Use Committee (Hungary) and Tierversuchskommission am Bundesministerium für Wissenschaft und Forschung (Austria).

### Subjects

All wolves that participated in this study were hand-raised by humans after being separated from their mothers in the first 10 days after birth. They were bottle-fed and later hand-fed by humans, and spent at least the first 3–4 months of their life in close human contact in the house of the hand-raiser handler, and regularly met strangers. They showed no fear of the testing apparatus or the experimenter.

In Study 1 we tested 8-week-old hand-raised dogs (N = 8, 5 males and 3 females, from three litters, mongrels) and hand-raised gray wolves (N = 13, 7 males and 6 females, from six litters).

In Study 2 we tested 4-month-old pet dogs (N = 7, 4 males and 3 females, mean age = 3.78 months, different breeds) and hand-raised gray wolves (N = 7, 5 males and 2 females, mean age = 3.71 months, from three litters). The wolves lived in two different wolf parks; four at Horatius Wolf Park, Gödöllő, Hungary (these had already been tested once in Study 1), and three at Wolf Science Center, Grünau, Austria.

In Study 3 we tested adult pet dogs (N = 8, 5 males and 3 females, mean age 3.25 years, different breeds) and hand-raised gray wolves (N = 8, 4 males and 4 females, mean age: 4.5 years, from three litters, kept in Horatius Wolf Park, Gödöllő). After the age of 3–4 months they lived in packs in large enclosures, and sometimes participated in public shows and/or film shootings.

In order to balance the animals' hunger state in the compared groups, we applied some restrictions in the feeding regime of the subjects. All tests in Study 1 and 2 were carried out in the morning. The 8-week-old puppies were last fed 1.5 hours prior to the test and the 4-month-old subjects were last fed during the previous evening. Adult subjects were tested in different times of the day. Adult dogs had their last meal on the day before the test, and adult wolves were tested about 1–1.5 days after the last feeding (since they ate large quantities on one occasion).

### Experimental arrangement

In Study 1 and 2 both the wolves and the dogs were tested in a room. In Study 3 adult dogs were tested in a room and wolves (except one) were tested in a familiar, quiet open-air place. Two plastic bowls were used for hiding the bait. As bait we used small pieces of cold cut for the dogs and raw meat for the wolves. Both bowls were extensively scented with the food before each experiment. The two bowls were placed 1.3 m apart in Study 1 and 1.5 m in Study 2 and 3. The experimenter (E) stood 20–30 cm behind the bowls on the middle line between the pots. (In Study 1 E was kneeling during the pointing because of the smaller height of the subjects.) The subject and the owner/handler stood facing the experimenter at a distance of 2.5 m.

### Procedure

The procedure was basically the same as described in Virányi et al. [12]. The subjects were held on a leash by their owner/handler and released after the pointing gesture was presented by a trained E. The tests were video recorded and analysed later.

We carried out four warm up trials to assess motivation and let the subjects learn that they can find food in the bowls. In these trials, the experimenter showed a piece of food to the subject and dropped it into one of the bowls. The owner released the subject, and it was allowed to eat the food if it chose correctly. This procedure was repeated twice for each bowl prior to the test session, and once on each side before control trials in the adult wolves. If the subject went to the wrong bowl in a warm up trial, the trial was repeated once. Subjects that did not choose a bowl more than twice and/or did not eat the food during the warm up trials were not tested.

In the test trials the subject could not see the hiding since the experimenter held the bowls in front of her chest and turned away from the subject while putting the bait into one of the bowls. After placing the bowls on the floor she stood with hands bent in front of her chest and tried to establish eye contact with the subject. If needed, she called the subject by its name or clapped with her hands. As soon as eye contact was established, the experimenter enacted the pointing gesture and kept looking at the subject till it made its choice. If the subject did not choose after releasing (it did not leave the start point or went to the E), eye-contact was re-established and the gesture was repeated. The order of the baiting was counterbalanced and randomized with the restrictions that one side could be rewarded only twice in a row and not on the very first two trials.

Our previous experiences with wolf pups showed that at the age of 8 weeks the pups' restricted visual field and visual motor coordination prevented them from processing the distal version of the gesture. Thus, in Study 1, we presented the 8-week-old subjects with 10 proximal momentary pointing trials, which is a simpler form of the task, enacted by a kneeling E. The main goal of Study 1 was to reveal the behavioural differences in the two species when the subjects had to collaborate with a human in a communicative situation at a very early age. (Actually, we have data on a larger dog sample in an independent study that dogs already at the age of 2 months are able to pass the distal momentary pointing test at the group level [17].

From the age of 4 months on, the critical *distal momentary* pointing gesture was used. To ensure optimal conditions, 4-month-old subjects received 14 trials, while adult individuals were presented with 20 trials.

In the *proximal momentary* trials (Study 1), E enacted a short (1 s) definite pointing with an extended index finger toward the baited bowl. The distance between the tip of the pointing finger and the bowl was about 30 cm. Only after the experimenter's hand returned to the starting position at her chest, was the subject released and allowed to make a choice. The *distal momentary* pointing (Study 2 and 3) was the same short signal with the only difference, that the distance between the pointing finger and the baited bowl was more than 50 cm **(Video S1)**. After a correct choice, the subject was allowed to eat the food, after an incorrect choice the baited bowl was lifted and the subject was not rewarded.

In Study 3, six control trials were carried out for 6 wolves in a separate session after the test. Although in case of dogs and young wolves, earlier studies provided evidence that olfactory cues did not affect the performance [18], [12], we wanted to exclude this possibility also in the case of adult wolves. These trials were conducted in the same way as the test trials but after E attracted the attention of the animal, she stood still and held her hands at her chest for one second while looking at the subject. Then the subject was allowed to make a choice.

### Variables and data analysis

One-sample *t*-tests were applied to compare the success against chance performance. The success of the groups of same aged dogs and wolves were compared by independent *t*-tests. (In order to compare the success of the different groups, we present the percentage of the correct choices on the figures.)

Three behaviour variables were coded: The *latency of eye-contact* with the E was measured as the time elapsed from the E's first attention getting action until the subject established eye contact with E and watched her pointing gesture. The *duration of struggling* included behaviours showing the subject's resistance to be controlled by the owner and wait for the human signal as well as its attempts to get free; in case of puppies this included lifting forelegs from the ground and/or turning the head back and forth, and in 4-month-olds and adults, pulling the leash or jumping. The *occurrence of biting* the hand of the handler was separately coded (**Video S2**). Latency, struggling and biting data were analysed using Mann-Whitney tests, since the data had different distributions and variances in the two groups. The behavioural data of one adult wolf could not be analysed due to problems with the video tape. The behaviour of the three age-groups was compared with Kruskal-Wallis test. Though four wolves participated both in study 1 and 2, their data were included in the analyses, because minimal effect of learning during one short session (10 trials) could be assumed [see 12], the experimenters and the test locations were different, and there was a long interval between the tests (2 months). The associations among the variables were analysed by Spearman rank correlations.

## Results

### Study 1 – Proximal momentary pointing at the age of 8 weeks

In the first session, we could complete the 10 proximal momentary trials only with 6 of the 13 wolf pups. Six pups were excluded during the warm up session, because we either could not place or hold them on the starting point or they did not choose the pot where the E had dropped the bait. From the 9 dog puppies only one was excluded during the warm up session, because it did not eat the bait. Moreover, one wolf and one dog stopped choosing after a few trials in the test session. We tried to test the excluded subjects once again a week later. We still could not perform the test with 4 wolves and the dog, thus finally the results of 9 wolf pups and 7 dog puppies were analysed.

The wolf pup group performed at 66% (0.53 SE), which was above chance level (t_(8)_ = 3.12, p = 0.017), while the dog puppy group had 61.4% (0.51 SE) success (t_(6)_ = 2.2498, p = 0.066). For the pups that could be tested there was no difference in the success of the two groups (t_(14)_ = 0.608, p = 0.553), however, this conclusion is mitigated by the fact that over half of the wolf pups had to be excluded from this test.

### Study 2 – Distal momentary pointing at the age of 4 months

**Figure 1:**
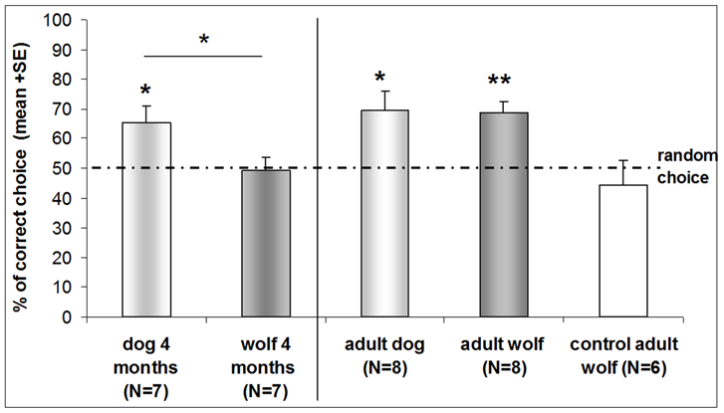
Study 2 and 3: The performance of young and adult wolves and dogs in distal momentary pointing trials. In all groups, success was compared to random choice (50%) with one sample t test. * P<0.05, ** P<0.01.

In the distal momentary trials, 4-month-old wolves performed at chance level (t_(6)_ = −0.135, p = 0.897). The performance of the dogs, however, was better than chance (t_(6)_ = 2.65, p = 0.038). There was a significant difference between the results of the two groups (t_(12)_ = 2.19, p = 0.049) **(** **Fig. 1** **)**.

### Study 3 – Distal momentary pointing in adulthood

The performance of both the adult dogs and wolves was better than chance (dogs: t_(7)_2.887, p = 0.023 and wolves: t_(7)_ = 5, p = 0.002). There was no significant difference between the success of the two groups (t_(14)_ = −0.081, p = 0.936). In the control trials the success of wolves did not differ from random choice (t_(5)_ = −0.663, p = 0.537) **(** **Fig. 1** **)**.

### Behavioural analyses

**Figure 2:**
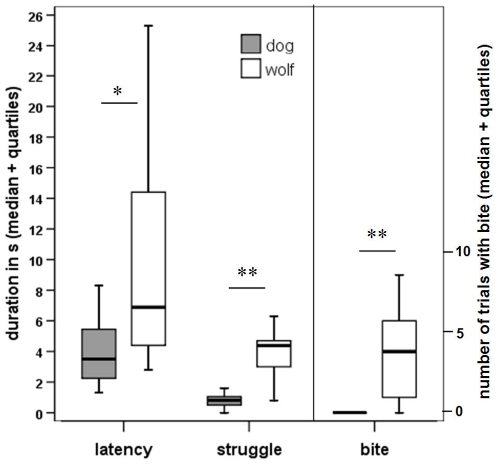
Study 1: The behaviour of 8-week-old, hand-reared dogs (N = 7) and wolves (N = 9) in the proximal momentary pointing test. * P<0.05, ** P<0.01.

We found significant differences between the wolf and dog groups in all coded behaviour variables at all three ages. Wolves needed more time than dogs to establish eye-contact with the pointing human (Study 1: Z = −2.064, p = 0.039; Study 2: Z = −2.503, p = 0.012; Study 3: Z = −2.546, p = 0.011). Wolves also struggled more with the handler than the dogs (Study 1: Z = −2.966, p = 0.003; Study 2: Z = −2.035, p = 0.042; Study 3: Z = −2.747, p = 0.006). In Study 1 wolf pups bit the handler more often than dog puppies (Z = −2.607, p = 0.009). In case of the 4-month-old and adult groups, none of the subjects tried to bite the handler (**Fig. 2** **–** **3** **)**.

**Figure 3:**
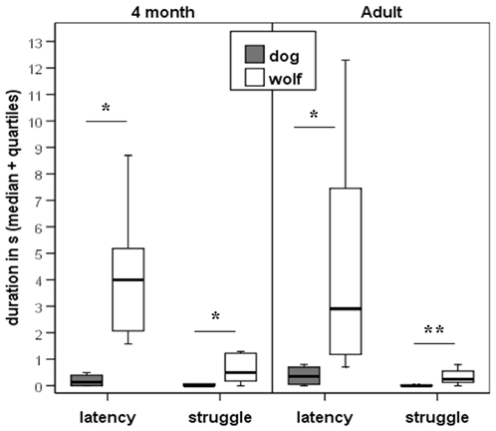
Study 2 and 3: The behaviour of 4-month-old and adult pet dogs (N = 7 and N = 8) and hand-raised wolves (N = 7 and N = 7) in the distal momentary pointing test. * P<0.05, ** P<0.01.

Although the latency of eye contact and the duration of struggling was always higher in the wolf groups than in the same-aged dog groups, in wolves the duration of struggling changed with age (H_(2)_ = 11.154, p<0.004) and latency had similar a tendency (H_(2)_ = 5.63, p = 0.059). The same age dependent difference was observed in the dogs (latency: H_(2)_ = 10.111, p = 0.006; struggle: H_(2)_ = 10.12, p = 0.006).

Neither the latency of eye contact nor the time of struggling was associated with success in any of the 8-week-old and 4-month-old groups, and in case of the adult dogs. However, adult wolves with lower eye-contact latency were more successful in the task (r_(7)_ = −0.86, p = 0.014).

In none of the dog groups did the time of struggling correlate with the latency of eye contact, however, wolves that struggled more had longer latency at the age of 8 weeks (r_(9)_ = 0.698, p = 0.036) and 4 months (r_(7)_ = 0.786, p = 0.036).

## Discussion

In the present study, we found age-dependent differences between wolves and dogs in the success to utilize human pointing and in their willingness to cooperate with the experimenter. The latter included differences in struggling and biting when held at a fixed position and attentiveness to a human experimenter. These detailed behavioural analyses offer a novel approach in pointing tests, and help to reconcile previously contradicting views on the effects of evolutionary and developmental processes.

At the age of 8 weeks, it was very difficult to make wolves attend to the human signal despite facing a relatively simple form of the task, namely proximal pointing. Even those wolf pups that could be tested had a higher latency for eye contact with the pointing E, struggled in the hands of the handler and bit her more often than dogs of the same age. The success of the two groups did not differ at this age. These results suggest that the tameness of the subjects and attention paid to the human experimenter do not influence the usage of this simple gesture. This is supported by the findings that socialized fox cubs are also able to utilize similar type of pointing gestures independently of having been selected for tameness or not [5].

In Study 2, using the more demanding, distal momentary pointing gesture, we found a marked difference in the performance of 4-month-old wolves and same-aged dogs. Importantly, these results confirmed previous findings that at this age only dogs are able to rely on this signal [11], [12], [17]. Though higher attentiveness was paralleled with increased willingness to cooperate in wolves by this age, the results show that even early and intensive socialisation of dogs and wolves in human environment is not sufficient to diminish differences in the performance in distal momentary pointing, as it has been suggested recently [8].

In Study 3, we provided evidence that socialised adult wolves are as successful in relying on *distal momentary* pointing as adult pet dogs. Adult wolves' success was paralleled with minimal struggling (and no biting) and high variability in the latency of eye contact at the group level. Importantly, success at the individual level in wolves correlated with the readiness to look at the pointing human. Dogs seemed to show a ceiling effect in this respect and this may explain the lack of correlation in their case.

The behavioural changes in wolves that paralleled the success in utilizing human distal pointing seem to support the hypotheses arguing for indirect selection during domestication [5], [11]. It seems, however, that selection for decreased levels of fear and aggression toward humans, as proposed by the emotional reactivity hypothesis, may be insufficient in accounting for higher interest in and cooperation with humans [5]. In addition a recent study revealed that selection for two factors under genetic influence (visual cooperation and focused attention) may have led independently to increased comprehension of human communicational cues in dogs [16]. Thus, the tendency for looking at humans in a communicative situation seems to be a genetic predisposition in dogs, while it is difficult to induce this behaviour in young wolves even after intense socialization [11]. However, intensive socialization could “mimic” the evolutionary effect at the individual level in wolves by lowering emotionality and leading to increased performance in some human controlled communicative situations.

Observations in an operant learning context [19] suggest that, compared to wolves, dogs have a better control of the suppression of immediate drives in favour of delayed rewards and show higher attentiveness to humans already at the age of 9 weeks. These differences give dogs a head-start in utilising human gestural signals, while delaying similar performance even in hand-reared and extensively socialised wolves.

We agree with Udell et al. [8] that in adult wolves an alternative route, predominated by extensive learning experiences about humans, can lead to similar performance in some human pointing tasks. In nature, during maturation wolves learn to take into account the behaviour of their pack mates in a feeding context. In addition, for being effective in this test, wolves have to learn about humans as social partners. This does not need to be in a special context, such as observing human visual gestures, but rather a general understanding that humans can provide useful information.

However, due to their less specific species recognition system and unique attachment behaviour, dogs are at an advantage to include humans in their social environment, and even intensively socialized wolves do not regard their caretakers as attachment figures [15]. This indicates that despite similar amount of early social interaction the role of humans as social partners is different in wolves and dogs. This is supported by results of Study 3, in which adult wolves still struggled significantly more and had longer eye-contact latency than dogs, though this difference was already relatively small at this age. This indicates that both learning processes described above have taken place during the first 3 years, and individuals who modulate their agonistic behaviour and cooperate with humans as social partners, performed indistinguishable from dogs in this task.

Note, that although in the present study we did not find differences in the success of socialized adult dogs and wolves, it does not necessarily follow that the ability of socialized wolves and dogs is the same with respect to other instances of communication with humans. Adult dogs are able to rely on even more demanding pointing types, which require the ability to generalize among contexts (cross body and asymmetric pointing: [2], [20]), or lack any discriminative component [21]. It may well be the case that wolves should reach a threshold in the latency/duration of attention, and then they can solve a given task. There could be different thresholds for different types of tasks. Dogs could be at an advantage in more complex tasks in social contexts, and further studies applying more subtle tests should be necessary to reveal such potential effects.

In sum, in dogs the necessary social skills for utilizing human pointing signals or the preparedness for their rapid development have been selected for in the domestication process. For wolves, a compensating developmental route might enable the establishment of the behavioural basis of successful communication and cooperation with humans in some tasks. Wolves, however, react to a lesser degree to socialisation in contrast to dogs, which are able to display control of agonistic behaviours and inhibition of actions in a food related task early in development. The synergistic hypothesis suggests that the dog-wolf difference in the sensitivity for human gestural cues emerges both at the evolutionary and developmental level. Further studies are needed to investigate whether this can be interpreted in the phenotype as a developmental change in the timing (heterochrony) of some social skills in dogs.

## Supporting Information

Video S1 Behaviour Variables. The video illustrates the behaviour variables (latency of eye-contact, struggling, biting) and the behaviour of 8-week-old wolves in the proximal momentary pointing trials in Study 1.
(9.04 MB AVI)

Video S2 Distal Momentary Pointing. The video illustrates the procedure of the distal momentary pointing and the behaviour of adult wolves in Study 3.
(9.92 MB AVI)
