# Correction: Explaining Dog Wolf Differences in Utilizing Human Pointing Gestures: Selection for Synergistic Shifts in the Development of Some Social Skills

**DOI:** 10.1371/annotation/9d7a0174-3068-4c44-bb98-b8a9bc5a99d5

**Published:** 2009-09-24

**Authors:** Márta Gácsi, Borbála Győri, Zsófia Virányi, Enikö Kubinyi, Friederike Range, Beatrix Belényi, Ádám Miklósi

The second author's name is incorrect. The correct name is: Borbála Győri. The correct citation is: Gácsi M, Győri B, Virányi Z, Kubinyi E, Range F, et al. (2009) Explaining Dog Wolf Differences in Utilizing Human Pointing Gestures: Selection for Synergistic Shifts in the Development of Some Social Skills. PLoS ONE 4(8): e6584. doi:10.1371/journal.pone.0006584

